# High Conservation of Tetanus and Botulinum Neurotoxins Cleavage Sites on Human SNARE Proteins Suggests That These Pathogens Exerted Little or No Evolutionary Pressure on Humans

**DOI:** 10.3390/toxins9120404

**Published:** 2017-12-19

**Authors:** Stefan Carle, Marco Pirazzini, Ornella Rossetto, Holger Barth, Cesare Montecucco

**Affiliations:** 1 Institute of Pharmacology and Toxicology, University of Ulm Medical Center, Albert-Einstein-Allee 11, 89081 Ulm, Germany; holger.barth@uni-ulm.de; 2 Department of Biomedical Sciences, University of Padova, Via Ugo Bassi 58/B, 35131 Padova, Italy; ornella.rossetto@gmail.com (O.R.); cesare.montecucco@gmail.com (C.M.); 3 Institute for Neuroscience, National Research Council, Via Ugo Bassi 58/B, 35131 Padova, Italy

**Keywords:** ExAC, gnomAD, botulinum neurotoxin, tetanus neurotoxin, SNAP-25, VAMP-1/2, syntaxin-1A/1B

## Abstract

The Genome Aggregation Database presently contains >120,000 human genomes. We searched in this database for the presence of mutations at the sites of tetanus (TeNT) and botulinum neurotoxins (BoNTs) cleavages of the three SNARE proteins: VAMP, SNAP-25 and Syntaxin. These mutations could account for some of the BoNT/A resistant patients. At the same time, this approach was aimed at testing the possibility that TeNT and BoNT may have acted as selective agents in the development of resistance to tetanus or botulism. We found that mutations of the SNARE proteins are very rare and concentrated outside the SNARE motif required for the formation of the SNARE complex involved in neuroexocytosis. No changes were found at the BoNT cleavage sites of VAMP and syntaxins and only one very rare mutation was found in the essential C-terminus region of SNAP-25, where Arg198 was replaced with a Cys residue. This is the P1’ cleavage site for BoNT/A and the P1 cleavage site for BoNT/C. We found that the Arg198Cys mutation renders SNAP-25 resistant to BoNT/A. Nonetheless, its low frequency (1.8 × 10^−5^) indicates that mutations of SNAP-25 at the BoNT/A cleavage site are unlikely to account for the existence of BoNT/A resistant patients. More in general, the present findings indicate that tetanus and botulinum neurotoxins have not acted as selective agents during human evolution as it appears to have been the case for tetanus in rats and chicken.

## 1. Introduction

A large effort is underway to catalogue variation in human genome and exomes (protein-coding regions of genes) in order to identify human mutations and their relation to a given pathological phenotype [1,2,3]. The largest database presently available is provided by the Exome Aggregation Consortium (ExAC) that stores in a publicly accessible library a data set spanning >60,000 exomes of European, African, South Asian, East Asian, and admixed American (Latino) ancestry [3] (http://exac.broadinstitute.org/about). More recently, this database has merged into the Genome Aggregation Database (gnomAD) raising the number of exomes to more than 120,000 and including about 16,000 genomes collected from various disease-specific individuals and population genetic studies (http://gnomad.broadinstitute.org/about). This has greatly increased the significance of finding mutations.

Whole exome/genome sequencing data have already provided very useful information and have led to the remarkable evidence that humans can carry known pathogenic mutations without being affected by the previously reported and associated disease [3,4,5,6]. Here, we have considered how human mutations can impact on the possibility of being affected by tetanus and botulism. These two neuroparalytic diseases are caused by the most potent bacterial exotoxins known to humans, i.e., tetanus and botulinum neurotoxins (TeNT and BoNTs), that enter nerve terminals and display a very specific metalloprotease activity directed to the three SNARE (Soluble NSF Attachment Protein Receptor) proteins VAMP (vesicle associated membrane protein, also known as synaptobrevin), SNAP-25 (synaptosomal-associated protein 25) and syntaxin (STX) [7,8]. These three proteins contain α-helical SNARE motifs that are capable of coil-coiling with each other forming the SNARE complex which is the core of the nanomachine mediating synaptic vesicle fusion with the plasma membrane [9]. Each neurotoxin cleaves a specific SNARE protein at a distinct peptide bond and prevents the release of neurotransmitters causing a reversible peripheral neuroparalysis [10,11,12]: TeNT and BoNT/B cleave the Q76(P1 site)-F77(P1’ site) (human numbering from here following) peptide bond of VAMP-2 [13], BoNT/D and /DC cleave the K59-L60 bond [14,15], BoNT/F1 cleaves the Q58-K59 bond [16], BoNT/G cleaves the A81-A82 bond [17]. More recently it was found that BoNT/F5 and BoNT/FA (also known as BoNT/H) hydrolyses the L54-E55 bond of VAMP-2 [18,19], and BoNT/X cleaves R66-A67 [20]. The BoNT-like protease of *Weissella oryzae* cleaves the W89-W90 peptide bond of VAMP [21]. BoNT/A cleaves the Q197-R198 bond at the C-terminus of the second SNARE motif of SNAP-25, whereas BoNT/E hydrolyses the R180-I181 peptide bond of the same protein [14,22,23,24]. BoNT/C is unique because it hydrolyses both SNAP-25 (at R198-A199) and STX-1A (at K253-A254) as well as STX-1B (at K252-A253) [25]. Such molecular lesions cause a persistent inhibition of neurotransmitter release with the consequent spastic neuroparalysis of tetanus and the flaccid neuroparalysis of botulism [26]. The long duration (3/4 months) of the BoNT/A induced paralysis in humans and its complete reversibility is at the basis of its large use in human therapy and cosmesis [10].

Previously, rats and chickens were found to be resistant to tetanus because VAMP-1, which is the predominant form of VAMP in the spinal cord, is mutated at the TeNT cleavage site [27]. In addition, evidence was provided recently that BoNT/D may have acted as a selective agent in animal botulism, and it was suggested that the presence of Ile at position 48 of VAMP-1 in place of Met is responsible for the resistance of humans to BoNT/D [28,29]. Using the ExAC and gnomAD databases, we tested whether human SNARE proteins display increased variability at the cleavage sites of TeNT and BoNTs which would indicate that these neurotoxins may have acted as selective agents driving the evolution of human SNARE proteins. At the same time, we tested the possibility that mutation at the SNAP-25 Q197-R198 peptide bond contributes to the resistance to BoNT/A therapy of some patients. Interestingly, we only found a mutation in Arg198 of SNAP-25, which is the P1’ site of cleavage by BoNT/A and, at the same time, the P1 site of cleavage by BoNT/C. Here, we show that the mutant SNAP-25 is resistant to the metalloproteolytic activity of BoNT/A. Remarkably, no mutations were found in VAMP-1 and -2 at the proteolytic sites of TeNT and BoNT/B, /D, /F, /G and /X or at those of BoNT/C on syntaxins 1A and 1B. We also found a rare replacement of Arg68 of VAMP-1 which is at the cleavage site of BoNT/X. As BoNT/X has never been found to cause botulism in humans, this finding has not been considered further in the present work. The biological and toxicological implications of these findings are discussed.

## 2. Results

### 2.1. Mutants of the Neuronal SNARE Proteins in Humans

**Figure 1:**
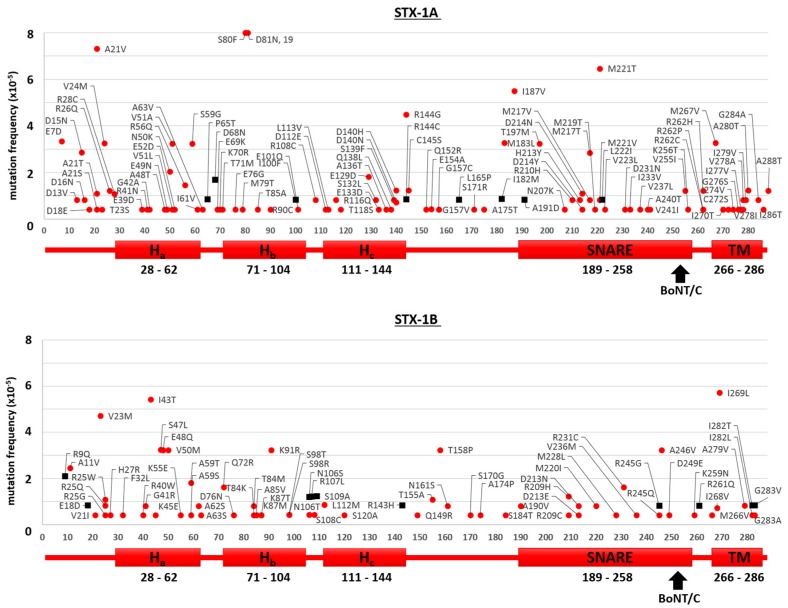
**Mutation frequency maps of human Syntaxin-1A/1B**. The figure displays the sequences of STX-1A (**top**) and STX-1B (**bottom**) with respective mutations found in the gnomAD database (red dots) together with those exclusively reported in the ExAC database (black squares). Only changes with respect to the canonical sequence of the corresponding protein are shown. Below each diagram a schematization of the protein primary structure and of its domains is given. Arrows indicate the cleavage sites by BoNT/C occurring at K253-A254 of STX-1A and at K252-A253 of STX-1B. No mutations within these peptide bonds were found.

**Figure 2:**
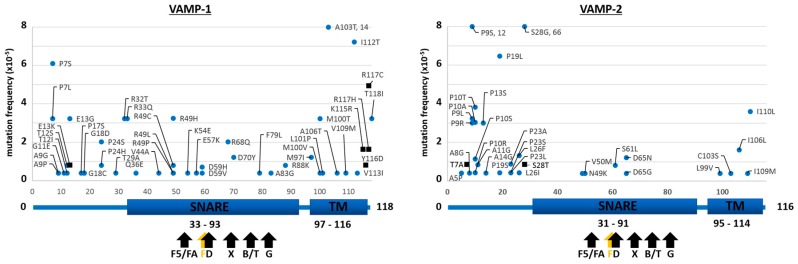
**Mutation frequency maps of human VAMP-1/2.** The figure displays the sequences of VAMP-1 (**left**) and VAMP-2 (**right**) with respective mutations found in the gnomAD database (blue dots) together with those reported exclusively in the ExAC database (black squares). Only changes with respect to the canonical sequence of the corresponding protein are shown. Frequency values higher than 8 × 10^−5^ are reported in the graph. Below each diagram a schematization of the protein primary structure and of its domains is given. Arrows indicate the proteolytic sites by the indicated neurotoxins: in VAMP-1 BoNT/F5 and BoNT/FA (L56-E57), BoNT/F (Q60-K61), BoNT/D and /DC (K61-L62), BoNT/B and TeNT (Q78-F79) and BoNT/G (A83-A84); R68 at the cleavage site of BoNT/X in VAMP-1 is substituted by Q; in VAMP-2 BoNT/F5 and BoNT/FA (L54-E55), BoNT/F1 (Q58-K59), BoNT/D and /DC (K59-L60), TeNT and BoNT/B (Q76-F77) and BoNT/G (A81-A82).

**Figure 3:**
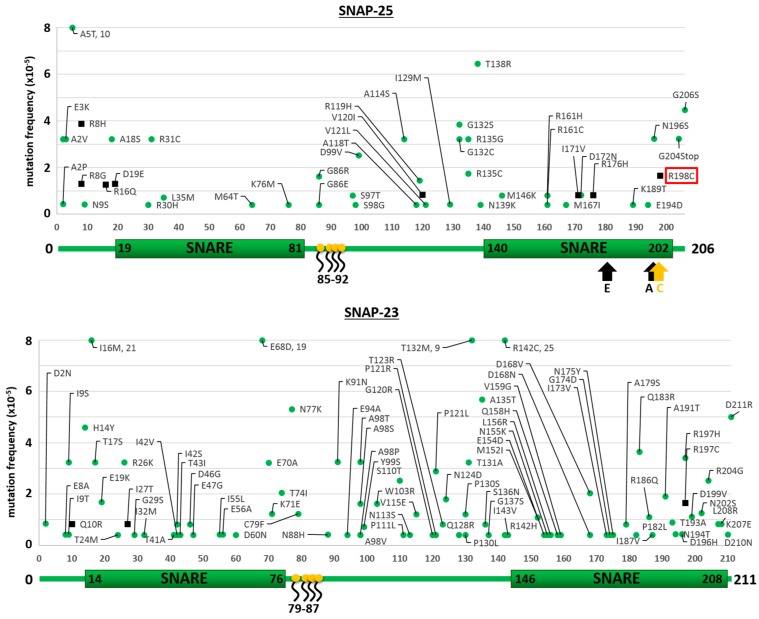
**Mutation frequency maps of human SNAP-25 and SNAP-23 proteins**. The figure displays the sequences of SNAP-25 (**top**) and SNAP-23 (**bottom**) with respective mutations found in the gnomAD database (green dots) together with those reported exclusively in the ExAC database (black squares). Only changes with respect to the canonical sequence of the corresponding protein are shown. Mutations with frequency higher than 8 × 10^−5^ are reported in the graph. Below each diagram a schematization of the protein primary sequence, of its domains and of the four palmitoylated cysteines (in yellow) is given. Arrows indicate the cleavage site of indicated neurotoxins. BoNT/E cleaves at R180-I181, BoNT/A cleaves at Q197-R198 peptide and BoNT/C cleaves at R198-A199. The red box indicates the R198C mutation.

The results of the searches in the ExAC and gnomAD databases for the neuronal isoforms of syntaxins (STX-1A and STX-1B), of VAMPs (VAMP-1 and VAMP-2) and SNAPs (SNAP-25 and SNAP-23) are reported in Figure 1, Figure 2 and Figure 3, respectively. We considered here only the protein-coding genetic variants (translated region) leading to mutations with respect to the canonical sequence of the corresponding protein. The first general comment is that, in comparison to proteins known to carry high sequence variability without functional consequences, e.g., major histocompatibility complex proteins (HLA-A/B/C) or olfactory receptors, the number of amino acid substitutions in SNAREs is dramatically lower, suggesting that they do not tolerate changes which would inevitably lead to functional alterations [30]. This indicates the very essential nature of their role in the physiology of the nervous system, so much so that even minor variations probably render the human carrier non-viable. Further, in agreement with the central role of the SNARE motif [31,32] is the finding that the few mutations identified are almost invariably outside the essential SNARE motif. However, some SNARE isoforms seem to tolerate a higher number of mutations, with SNAP-23, VAMP-1 and STX-1A displaying a higher number of mutants than SNAP-25, VAMP-2 and STX-1B. The reason for this difference is not apparent as their knock out in animal models lead to non-viability, apart for the STX-1A knock out mice [33,34,35,36,37]. Accordingly, Figure 1 shows that STX-1A may tolerate many mutations.

Importantly, it should be noted that mutants of neuronal SNAREs are characterized by an extremely low frequency in the population of the present data bases, and are never present in homozygosis indicating that these variations are likely deleterious. Thus, they should be considered as “mutations” and not “polymorphisms” [38]. This suggests that individuals carrying these mutations are very likely to be affected by pathological alterations, even though the phenotype is not made available on the databases. This possibility is consistent with available evidence linking alterations of SNARE proteins to human neuropsychiatric disorders, like epilepsy, schizophrenia and autism [39,40,41], and to significant pathological consequences both in human and in animal models [42,43,44].

### 2.2. Mutations of Human SNARE Proteins at the Cleavage Sites of Tetanus and Botulinum Neurotoxins

Figure 1 and Figure 2 show that none of the residues forming the peptide bond cleaved by TeNT or BoNT proteases within VAMP-1/2 and STX-1A/1B are mutated. This indicates that these very potent neurotoxins have not acted significantly as selective agents during human evolution. At variance, the red rectangle in the top panel of Figure 3 highlights a mutation in SNAP-25, where Arg 198 is replaced by Cys at the cleavage sites of BoNT/A and /C. This mutation is very rare as it has been detected in 2 out of >120,000 subjects and in both cases in heterozygosis. At variance from VAMPs and syntaxins, SNAP-25 and SNAP-23 are not integral proteins of the membrane and are also unique among SNARE proteins as they contain two SNARE motifs (Figure 3). In fact, SNAP-25 and SNAP-23 are attached to the cytosolic face of the plasma membrane or of intracellular organelles, like synaptic vesicles, via palmitoylated cysteine quartets (residues 85–92 and 79–87, respectively, in yellow) located in between the two SNARE motifs [45,46].

**Figure 4:**
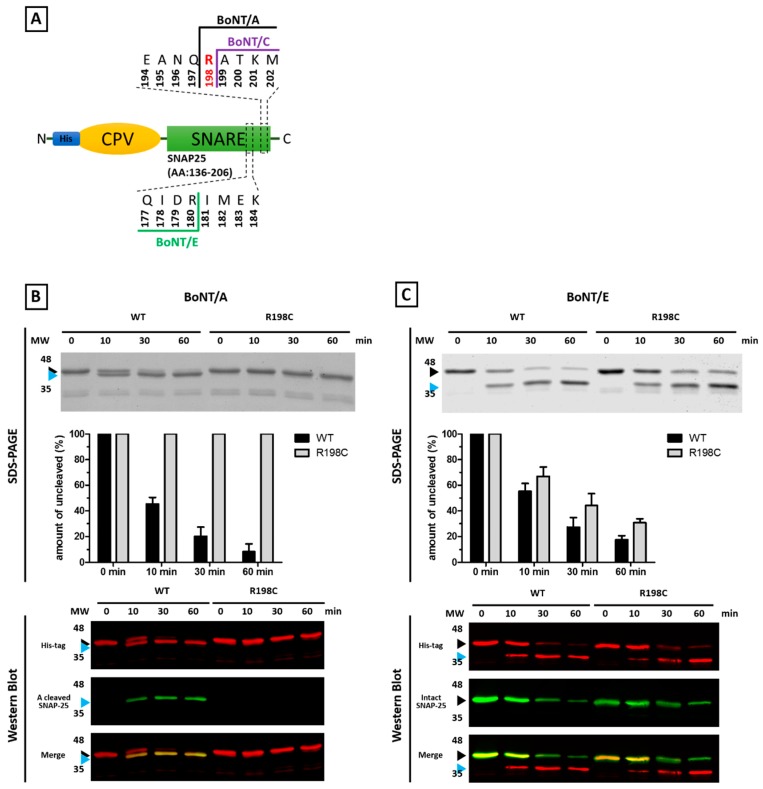
**Western blot analysis of BoNT/A and /E cleavage of wild type (WT) and mutant (R198C) SNAP-25 in vitro**. (**A**) Scheme of the fusion protein used to test SNAP-25 and SNAP-25 R198C cleavability. Recombinant SNAP-25 substrate (300 ng/sample) was incubated with reduced full length BoNT/A (**B**) or BoNT/E (**C**) (10 ng/sample). At indicated time points, the reaction was blocked by adding loading sample buffer and heat denaturation. Samples were separated by SDS-PAGE and proteins stained with Coomassie Blue. Ratio of uncleaved to total SNAP-25 was evaluated by densitometric analysis. Ratios are shown in the bar chart as mean with respective standard deviation of 3 independent experiments. In some experiments, after electrophoresis, samples were immunoblotted for His (recognizing both intact and truncated SNAP-25), BoNT/A-cleaved SNAP-25 or whole SNAP-25 (recognizing only native SNAP-25) detection. Antibodies were visualized using fluorescent secondary antibodies for detection by the Odyssey Imaging System. The assays were performed at least 3 times and representative blots are shown in the bottom panels.

R198 is the P1’ site of the peptide bond cleaved by BoNT/A. Because of the presence of the sulfur atom, the volume of the lateral chain of Cysteine is much larger and hydrophobic than that of Arginine, which is also positively charged at physiological pH values. Thus, it is very likely that Cysteine may not fit within the active sites of BoNT/A and /C, making SNAP-25 R198C resistant to these BoNTs. We tested this possibility by generating a recombinant protein consisting of an N-terminal His-tag followed by a CPV and the second SNARE motif of SNAP-25 (Figure 4A), which is the target substrate of BoNT/A. The protein was created both with the wild type sequence and the R198C mutation, and susceptibility to proteolysis by the neurotoxin was tested.

The SDS-PAGE analysis of Figure 4B shows that BoNT/A cleaves with high efficiency wild type SNAP-25, whilst the mutated recombinant SNAP-25, under these experimental conditions, is completely resistant. This result is even more evident in Western blot, using an antibody that specifically stains the C-terminus generated in SNAP-25 after BoNT/A proteolysis. Figure 4C also shows that the same mutation does not affect BoNT/E activity, as evidenced both by SDS-PAGE and Western blotting, using antibodies directed against the His-tag or against intact SNAP-25. This latter result suggests that the mutation does not impact on the folding of SNAP-25, and excludes the possibility that the lack of cleavage by BoNT/A derives from protein aggregation via disulfide bridging.

Since BoNT/C proteolyzes its substrates very poorly in the test tube [47] and the recombinant mutated SNAP-25 does not transfect efficiently in neurons in culture, the effect of mutation on BoNT/C activity could not be investigated. In addition, BoNT/C does not cause botulism in humans [7], and therefore it cannot have acted, in any case, as a selective agent for human SNARE proteins. However, considering that R198 is the P1 site of the peptide bond hydrolyzed by BoNT/C [48], we predict that this mutation will similarly impact on the cleavability of SNAP-25 by BoNT/C.

## 3. Discussion

Previous work indicated that TeNT and BoNT have acted to select mutants of the VAMP-1 protein, which are resistant to their proteolytic activity and therefore to the neuroparalysis induced by tetanus and botulism. It was found that rats and chicken are resistant to tetanus because VAMP-1 is the predominant isoform in the spinal cord and their VAMP-1 carry the peptide bond Val77-Phe78, not cleavable by TeNT, rather than the TeNT sensitive peptide bond Gln77-Phe78 present in most species, including humans [27]. The results reported here show no record of human mutants at any of the cleavage sites of the many BoNTs targeting neuronal VAMPs or syntaxins, indicating that these neurotoxins may have not acted as selective agents in human evolution. This result is expected for botulism, which is indeed a disease of animals with an accidental impact in humans [49,50]. The contrary was expected for tetanus, a disease that has been a major human killer during history, beginning with the descriptions of Hippocrates and Aretaeus [51,52], until the introduction of tetanus vaccine. Tetanus can occur at any age and it has been particularly evident among neonates in the horrible form of tetanus neonatorum, which has taken lives in the number of hundreds of thousand, and still is not completely eradicated [53,54].

The single mutation detected in a BoNT cleavage site in the present search in only two cases over >120,000 human subjects, is located in SNAP-25 at the residue Arg198 replaced by a cysteine residue. Even though we cannot validate such a mutation experimentally, the possibility of sequencing errors can be safely ruled out by the very stringent criteria by which data have been processed [3]. We provide evidence that this variation affects BoNT/A activity, and very likely that of BoNT/C, making SNAP-25 R198C resistant to these neurotoxins, at least under the experimental conditions we used. BoNT/A, like all the other BoNTs, is naturally present as many toxin variants, known as subtypes, and here we tested only subtype 1 [7,55]. Given that the BoNT/A variants characterized so far invariably cleave SNAP-25 at the Q197-R198, it is reasonable to assume that the present result may be extended to the other members of the BoNT/A family. However, this should be experimentally tested.

Cysteine Palmitoylation by Golgi S-palmitoyl transferases is a main determinant of SNAP-25 membrane association and trafficking to the plasma membrane [56,57]. Whether the additional Cysteine in the R198C mutant is palmitoylated in vivo and which functional consequences this modification may cause are difficult to predict. However, one might speculate that a further anchorage site to the plasma membrane at the C-terminus of SNAP-25 would sequester the second SNARE motif preventing its coil coiling with the other SNARE proteins. In this case, the mutation would further preclude the activity of BoNT/A as the cleavage of SNAP-25 by its protease relies on multiple enzyme-substrate interactions involving SNAP-25 C-terminus [7,10,58].

Finally, taking into account the number of genomes considered, the rarity of R198C mutation and the fact that no Cys198 homozygous subjects have been isolated so far, one can safely conclude that the human patients primary resistant to BoNT/A are not carrying mutations that prevent BoNT/A cleavage of SNAP-25.

## 4. Conclusions

The availability of human genome database has allowed us to check the possibility that the TeNT and BoNTs have acted as selective evolutionary agents in humans. This possibility is not supported by the present analysis as we have found no mutation in their specific cleavage sites which appear to be essential for the SNARE function. In fact, the present data indicate that tetanus and botulinum neurotoxins have been selected through bacterial evolution to act in such a way as to cause a major impairment to the very essential process of neurotransmitter release. Their cleavage of VAMPs and syntaxins leads to the removal from the membrane of a large portion of the molecule, which is no longer available to act within the membrane active neuroexocytosis nanomachine. The same explanation applies to BoNT/E which removes a large portion of SNAP-25, but not to BoNT/A which removes only nine residues from the SNAP-25 C-terminus. Several explanations have been advanced to account for the possible consequence of this truncation, but a satisfactory rationale has still to be provided.

## 5. Materials and Methods

### 5.1. Generation of SNARE Protein Mutation Maps

Mutation frequency tables of depicted proteins were obtained from http://www.exac.broadinstitute.org/ and http://gnomad.broadinstitute.org/. Schematic figures, including the protein domains were made using http://www.uniprot.org/.

### 5.2. Molecular Cloning

As recombinant target substrate for BoNT/A and BoNT/E, a His-tagged construct consisting of N-terminal CPV and C-terminal the second SNARE motif of SNAP25 (amino acids 136–206) connected by a GSG linker was designed. The constructs for wild type and R198C were synthesized by GeneArt Thermofisher and delivered in a donor vector. Using standard cloning techniques, the gene of interest was cloned into a pET28a expression vector by BamHI/HindIII restriction digestion and subsequent ligation.

### 5.3. Expression and Affinity Purification of Recombinant His_CPV_SNAP25(136–206) Wild Type and R198C

Expression plasmids were transformed into *E. coli* BL21. A single clone was picked and used for overnight culture at 37 °C and 180 rpm (Gallenkamp orbital shaker incubator) in 5 mL LB (10 g/L Tryptone, 5 g/L Yeast extract, 5 g/L NaCl, 15 g/L Agar) + Kanamycin (100 µg/mL) in a culture tube. The next day the total volume was transferred into 500 mL of LB + Kanamycin in a 1 L Erlenmeyer flask and further incubated at 30 °C and 220 rpm till the OD_600_ reached 0.5–0.6. Expression was then induced using 0.5 mM IPTG and the culture was further incubated at 30 °C and 220 rpm overnight. *E. coli* was harvested by centrifuging for 15 min at 5500 RCF. The pellet was solubilized in 10 mL Binding Buffer (20 mM Tris-HCl, 500 mM NaCl, pH 7.8) and frozen until use. For purification the pellet was thawed and substituted with Roche complete protease inhibitor cocktail (EDTA free). The solution was sonicated in an ice-cooled falcon at 80% amplitude and 0.5 s cycles 8 times (30 s pauses between each time). The mixture was pelleted by centrifugation at 16,000 RCF for 30 min at 4 °C. The supernatant was then 0.22 µm filtered and adjusted to pH 7.8. Purification was performed with the ÄKTA FPLC system using a HiTrap Talon Crude 1 mL column and a standard flow of 1 mL/min. The system was flushed with binding buffer (5 CV). Sample was loaded and flow-through collected for an additional run. Subsequently, the system was washed with 15 CV of washing buffer (20 mM Tris-HCl, 500 mM NaCl, 5 mM Imidazole, pH 7.8). Gradient elution was performed from 0–100% with elution buffer (20 mM Tris-HCl, 500 mM NaCl, 500 mM Imidazole, pH 7.8) within 20 CV. Fractions were collected and analyzed using Coomassie gels. Product containing fractions were pooled and 3 steps dialyzed against Hepes buffer (10 mM Hepes, 120 mM NaCl, pH 7.4). Aliquots were stored at −80 °C.

### 5.4. BoNT Proteolysis Assay

BoNT/A was prepared and purified as described [59] whilst BoNT/E was produced in *E. coli* via recombinant methods [23]. BoNTs were reduced using the assay buffer (150 mM NaCl, 10 mM NaH_2_PO_4_, 0.3 mM ZnCl_2_, 15 mM DTT, complete protease inhibitor [EDTA free], pH 7.4) and incubation at 37 °C for 30 min. The BoNT solution was then mixed with assay buffer containing an appropriate amount of wild type or mutant substrate and incubated at 37 °C. The whole experiment was performed in a single test tube. At the indicated time points, samples were taken from the test tube and the reaction was stopped by adding 6× protein loading buffer and incubation at 95 °C for at least 5 min. The residual mixture was further incubated at 37 °C till all samples were taken and applied to SDS-PAGE and Western blot depending on the experiment.
